# Effects of Whey Protein Supplementation on Muscle Damage, Hepatic Stress, and Inflammatory Markers in Power‐ and Endurance‐Trained Athletes

**DOI:** 10.1002/ejsc.70279

**Published:** 2026-10-26

**Authors:** Mouna Ben Mhamed, Mounir Landolsi, Mehdi M'rad, Afef Bahlous, Faysal Zarrouk, Hejer Trabelsi, Katja Weiss, Thomas Rosemann, Ezdine Bouhlel, Beat Knechtle

**Affiliations:** ^1^ Higher Institute of Sport and Physical Education of Kef University of Jandouba Kef Tunisia; ^2^ Biology, Medicine, and Health Faculty of Medicine of Sousse Research Laboratory (LR19ES09) Exercise Physiology and Pathophysiology: From Integrated to Molecular University of Sousse Sousse Tunisia; ^3^ Laboratory of Clinical Biochemistry and Hormonology Pasteur Institute of Tunis University of Tunis‐El Manar Tunis Tunisia; ^4^ Higher Institute of Sport and Physical Education of Kssar Saïd University of Manouba Tunis Tunisia; ^5^ Institute of Primary Care University of Zurich and University Hospital of Zurich Zurich Switzerland

**Keywords:** hepatic stress, inflammatory response, muscle damage biomarkers, training modality, whey protein supplementation

## Abstract

This study investigated the effects of 8 weeks of whey protein supplementation (30 g/day) on biomarkers of liver function, inflammation, and muscle damage in trained power‐trained (predominantly speed‐power/anaerobic‐power disciplines) and endurance‐trained athletes, in a randomized, double‐blind, placebo‐controlled design. Fifty‐nine male athletes were stratified into power (*n* = 30) and endurance (*n* = 29) groups, then randomly assigned to a whey protein group (WG) or a placebo control group (CG). Biomarkers assessed pre‐ and post‐intervention included alanine aminotransferase (ALT), aspartate aminotransferase (AST), C‐reactive protein (CRP), and creatine kinase (CK). Significant reductions in ALT, AST, and CK were observed in both endurance‐ and power‐trained athletes after supplementation (*p* = 0.001, *p* = 0.015, *p* = 0.001, and *p* = 0.003 respectively), indicating attenuated hepatic and muscular stress. Among the four biomarkers, only ALT displayed a statistically significant three‐way interaction (Time × Supplementation × Training modality; *p* = 0.003), identifying it as the sole marker showing a genuine modality‐specific differential response to supplementation. A significant within‐group reduction in CRP was additionally observed in the supplemented power‐trained athletes (*p* = 0.039), whereas endurance athletes showed only minor, non‐significant changes; because the omnibus three‐way interaction for CRP was not significant, this finding is reported as a within‐group trend rather than evidence of a differential effect between modalities. Placebo groups demonstrated stable or increased enzyme and CRP values, particularly in endurance athletes. These findings suggest that whey protein may attenuate exercise‐induced hepatic and muscular stress across training modalities, with a modality‐specific effect on hepatic stress markers (ALT) observed in power‐trained athletes. The results highlight whey protein's potential as a nutritional aid to promote recovery and physiological adaptation, possibly mediated by antioxidant and anti‐inflammatory mechanisms. Further studies are warranted to examine long‐term outcomes and dose‐response effects across different training profiles.

## Highlights

- Eight weeks of whey protein supplementation (30 g/day) reduced hepatic (ALT, AST) and muscle (CK) enzymes in both power‐ and endurance‐trained athletes.
- The most pronounced modality‐specific effect was observed for ALT, the only biomarker showing a significant three‐way interaction (Time × Supplementation × Training modality); CRP showed a significant within‐group reduction in power‐trained athletes but no significant interaction across modalities.
- Placebo groups showed stable or increased enzyme and CRP levels, particularly in endurance athletes.
- Whey protein may act as a nutritional aid to attenuate exercise‐induced stress and promote recovery generally across training modalities, with a modality‐specific benefit demonstrated specifically for hepatic stress markers (ALT) in power‐trained athletes.
- Results highlight the need for individualized nutritional strategies according to the specific metabolic demands of the sport.

## 1 Introduction

High‐intensity physical training, whether performed in endurance or power disciplines, induces marked physiological alterations at the muscular, hepatic, and inflammatory levels, potentially disrupting homeostasis and impairing recovery and performance (Pasiakos et al. 2015; Peake et al. 2017). When exercise intensity exceeds the body's adaptive capacity, long‐term exposure may compromise essential tissue functions and contribute to overtraining (European College of Sport Science, and American College of Sports Medicine 2013). These disturbances are reflected in the elevation of biochemical markers such as creatine kinase (CK), alanine aminotransferase (ALT), aspartate aminotransferase (AST), and C‐reactive protein (CRP), indicating muscle damage, liver stress, and systemic inflammation. It should be noted that, although ALT and AST are conventionally used as hepatic markers, AST in particular is also abundantly expressed in skeletal muscle; post‐exercise elevations of both enzymes, but especially AST, may therefore partly reflect sarcolemmal leakage secondary to skeletal muscle damage rather than hepatocellular injury alone, a distinction addressed further in Section 4.1.

These biological markers are widely used to assess the degree of physiological stress and recovery following strenuous training (Zhao et al. 2015). In endurance athletes, prolonged and repetitive efforts frequently result in muscle microlesions and elevated oxidative stress, leading to low‐grade systemic inflammation, immune dysfunction, and progressive declines in muscle function (Nieman 2000). Many of these athletes engage in mixed training that includes eccentric exercises, plyometric drills, or obstacle‐based activities, which have been shown to intensify muscle damage and inflammatory responses (Hody et al. 2019; Peake et al. 2017). These alterations contribute to delayed recovery and may negatively impact subsequent performance (Peake et al. 2017).

Conversely, power‐trained athletes are frequently exposed to high mechanical loads and maximal muscle contractions, which provoke acute muscular trauma and initiate catabolic responses. This condition generates a heightened demand for efficient anabolic recovery mechanisms to restore muscle integrity and performance capacity. These divergent demands are expected to translate into differential biomarker responses to chronic whey intake: power‐trained athletes typically carry greater active muscle mass and rely predominantly on alactic/glycolytic energy pathways with high mechanical strain per session, favoring larger acute CK and transaminase excursions and, potentially, a more detectable anti‐catabolic benefit from leucine‐rich protein; endurance‐trained athletes instead accumulate damage through repetitive, lower‐force eccentric loading and oxidative metabolic stress, which may produce a more diffuse, lower‐amplitude inflammatory signature (e.g., CRP) that is harder to modulate detectably over 8 weeks (Fedewa et al. 2017).

In this context, nutritional strategies play a fundamental role in optimizing post‐exercise recovery, supporting tissue repair, and preventing chronic fatigue or injury. The post‐exercise ingestion of proteins, particularly those with high biological value and rapid absorption kinetics, has been shown to facilitate muscle protein synthesis. Whey protein, due to its high leucine content and digestibility, is particularly effective in stimulating anabolic pathways and may also exert antioxidant and anti‐inflammatory effects (Moore et al. 2014; Pasiakos et al. 2015; Phillips 2016; Tipton and Wolfe 2004). These characteristics suggest that whey protein could enhance recovery and reduce exercise‐induced stress in both endurance and power athletes (Bagheri et al. 2022; Davies et al. 2018).

Nevertheless, most available studies have focused on general athletic populations without distinguishing between training modalities, thereby limiting the interpretation of whey's effects across different physiological profiles. Few investigations have directly compared the biological responses to whey supplementation in endurance versus power athletes, despite their divergent metabolic and neuromuscular characteristics (Kritikos et al. 2021). Some studies report significant reductions in markers of muscle damage and inflammation following whey supplementation (Pasiakos et al. 2014), while others fail to show measurable effects, particularly when background dietary protein intake is already optimal (West et al. 2017).

Therefore, the present study aims to comparatively investigate the impact of 8 weeks of whey protein supplementation on muscle (CK), hepatic (ALT, AST), and inflammatory (CRP) biomarkers in power and endurance‐trained athletes. We hypothesize that post‐exercise whey protein intake will reduce exercise‐induced hepatic and muscular stress, with a potentially more pronounced effect in power‐trained individuals.

## 2 Materials and Methods

Given the physiological rationale and the hypothesis that whey protein supplementation may modulate hepatic, muscular, and inflammatory biomarkers differently in endurance and strength athletes, we designed a randomized, double‐blind, placebo‐controlled trial. The methodological framework was developed to ensure rigorous control of training load, diet, and environmental factors, thereby minimizing potential confounders and allowing reliable attribution of observed effects to the supplementation protocol.

### 2.1 Study Design

This investigation was conducted as an 8‐week randomized, double‐blind, placebo‐controlled study comparing the effects of whey protein supplementation between competitive, trained endurance and power athletes. The protocol was approved by the local Biomedical Ethics Committee (Approval No. 2022/12/E/V1) and was carried out in accordance with the Declaration of Helsinki. All participants received comprehensive oral and written information on study procedures, potential risks, and benefits before signing informed consent forms.

During the first week, baseline anthropometric measurements, medical examinations, and familiarization with laboratory equipment and procedures were conducted to ensure measurement reproducibility. Participants were then randomly assigned, within each training modality, to either a whey protein supplementation group (WG) or a placebo control group (CG) using a computer‐generated randomization sequence. Both athletes and researchers involved in data collection were blinded to group allocation.

### 2.2 Participants and Simple Size Estimation

A priori sample size calculation was performed using G*Power software (v3.1.9.4) for a mixed ANOVA (repeated measures, within‐between interaction). The input parameters were selected in accordance with Cohen's conventions and aligned with sports nutrition literature on muscle damage markers (Davies et al. 2018). Assuming a medium effect size (*f* = 0.25), a significance level *α* = 0.05, and a conservative correlation of 0.5 between repeated measures, a total sample size of 48 athletes was required to achieve a standard statistical power of 80%. Our final cohort of 59 completed participants provides an actual calculated statistical power of 88%, ensuring the study is sufficiently powered to detect true group × time interaction effects and mitigating the risk of Type II errors for secondary outcomes.

Fifty‐nine male track‐and‐field competitive, trained athletes, and aged 18–26 years, volunteered to participate and were divided into two cohorts based on their discipline: endurance‐trained (*n* = 29) and power‐trained (*n* = 30) training. All participants were actively competing at a national or international level and had been training regularly (≥ 5 sessions/week, 2 hours per session) for at least 6 months prior to inclusion in the study. Notably, under contemporary framework guidelines (e.g., McKay et al. 2022) framework), a 6‐month competitive background categorizes these participants as trained/developmental athletes rather than historically elite populations. Endurance athletes specialized in middle‐ and long‐distance events, whereas power‐trained athletes competed in disciplines requiring high mechanical power output (shot put, javelin, long jump, triple jump, pole vault, 100–400 m sprints).

Inclusion criteria were as follows: absence of musculoskeletal, respiratory, cardiovascular, or metabolic disorders, non‐smoking status, and no current use of performance‐enhancing substances. Participants were required to abstain from caffeine, alcohol, and medication for 48 h before testing sessions. Use of any protein supplements, antioxidants, creatine, or anabolic steroids was prohibited during the study.

All participants resided at the national training center throughout the experimental period, ensuring uniformity of diet, accommodation, and training conditions. Physical characteristics of each group (age, height, body mass, body mass index [BMI]) were recorded (Table 1) and verified for baseline equivalence. Within each training modality, athletes were randomized into whey (WG) and placebo (CG) subgroups using block randomization with a predefined 2:1 allocation ratio, stratified by competitive expertise (elite vs. club level), yielding four sub‐groups: power‐WG (*n* = 20), power‐CG (*n* = 10), endurance‐WG (*n* = 20), and endurance‐CG (*n* = 9), presented in Table 2 within a 2 × 2 (Training modality × Supplementation) layout.

**TABLE 1:** Baseline characteristics of endurance and Power athletes in the whey and placebo groups.

	Endurance athletes (EA)		Power athletes (PA)	
	WG (*n* = 20)	CG (*n* = 9)	WG (*n* = 20)	CG (*n* = 10)
Age (years)	18.2 ± 1.4	18.6 ± 4.0	19.1 ± 3.8	17.6 ± 1.5
Height (cm)	175.2 ± 4.9	174.4 ± 5.8	181.6 ± 5.3	173.5 ± 4.4
Body Mass (kg)	59.9 ± 6.0	62.1 ± 9.2	75.4 ± 9.9	72.0 ± 11.9
BMI (kg/m^2^)	19.5 ± 2.1	20.3 ± 2.1	22.9 ± 3.0	23.8 ± 3.0
Body fat (%)	13.18 ± 2.64	13.72 ± 2.10	15.95 ± 2.99	14.06 ± 4.98

Abbreviations: BMI: body mass index; CG: placebo control group; WG: whey group.

### 2.3 Training Program

Both endurance and power‐trained athletes maintained their usual training schedules, standardized at 5 sessions/week for 2 h/session, with intensity adapted to sport‐specific demands (Table 2). Endurance training intensity ranged from 60% to 110% of maximal aerobic velocity (MAV), while power‐trained athletes trained between 50% and 90% of maximal anaerobic power. Training loads were monitored to ensure consistency across the intervention period.

**TABLE 2:** Training program parameters for each athlete category during the experimental period.

	Endurance‐WG (*n* = 20)	Endurance‐CG (*n* = 9)	Power‐WG (*n* = 20)	Power‐CG (*n* = 10)
Hourly volume	5 × 2 h/week	5 × 2 h/week	5 × 2 h/week	5 × 2 h/week
Average session intensity (% of maximum possibilities)	60%–110% MAV	60%–110% MAV	50%–90% MAP	50%–90% MAP
Session frequency	5 times/week	5 times/week	5 times/week	5 times/week

Abbreviations: ETA: endurance trained athlete; MAP: maximum anaerobic power; MAV: maximum aerobic velocity; PTA: Power‐trained athlete.

### 2.4 Supplementation Protocol

The whey protein group received a daily post‐exercise supplement of 30 g whey protein powder (IronMaxx, Hürth, Germany; 100% Whey Protein, Latte Macchiato flavor), providing 197 kcal and a high leucine content. The supplementation was ingested within 30 min after training. The placebo control group received an isocaloric, isovolumetric carbohydrate‐based powder (∼30 g carbohydrate, ∼197 kcal) matched for appearance and taste. Supplementation compliance was monitored via individual intake logs completed daily by participants. Habitual dietary intake, including baseline protein consumption (g/kg/day), was not formally quantified via dietary recalls or food diaries in this study; diet was standardized only through communal catering at the national training center. We therefore cannot rule out that part of the observed biochemical improvements reflects correction of an underlying suboptimal baseline protein intake in some participants, rather than a specific ergogenic action of whey per se; nor can we exclude an independent insulinotropic effect of the carbohydrate‐based placebo on post‐exercise proteolysis. Both points are acknowledged as limitations in Section 4.5.

### 2.5 Anthropometric Measurements

At baseline, anthropometric assessments were performed by an experienced investigator in the morning, with participants in a fasted state, barefoot, and wearing light sportswear. Height was measured to the nearest 0.1 cm using a stadiometer (Seca, Basel, Switzerland), and body mass to the nearest 0.1 kg using a digital scale (Tanita 545 N, Tokyo, Japan).

Body fat percentage was estimated from four skinfold thicknesses (bicipital, tricipital, subscapular, suprailiac) measured with Harpenden calipers (Baty International, UK), following the protocol of (Durnin and Womersley 1969). Body density was calculated as

$$\text{Body}\;\text{density}=1.1765-0.0744\left(\log_{10}\sum S\right)$$

where ∑S is the sum of skinfolds in millimeters. Percent body fat was then determined using:

Bodyfat(%)=(4.95/bodydensity−4.5)×100

### 2.6 Biochemical Measurements

Venous blood samples (10 mL) were collected from the antecubital vein after an overnight fast, both before and after the supplementation period. The post‐intervention blood draw was scheduled no sooner than 72 h after each athlete's last training session and final supplement intake, in order to allow acute, session‐related elevations in CK, ALT, and AST (which typically peak between 24 and 72 h post‐exercise) to subside and thereby avoid confounding of the chronic 8‐week supplementation effect by transient acute‐exercise responses (Al Akko et al. 2024). Samples were processed for serum separation and analyzed for:

– Muscle damage marker: Creatine kinase (CK)
– Hepatic function markers: ALT and AST
– Inflammatory marker: C‐reactive protein (CRP)

Analyses were performed using a Cobas Integra 400 analyzer (Roche Diagnostics, Rotkreuz, Switzerland). All assays were conducted in duplicate, with intra‐assay variability < 5%.

### 2.7 Statistical Analysis

All statistical analyses were performed using SPSS software, version 16.0 (SPSS Inc., Chicago, IL, USA). Data normality was assessed using the Shapiro–Wilk test. A three‐way mixed‐design analysis of variance (ANOVA) was conducted, with Time (pre‐vs. post‐intervention) as the within‐subjects factor and Supplementation (whey vs. placebo) and Training modality (endurance vs. power) as the between‐subjects factors (2 × 2 × 2 design). Where significant main or interaction effects were observed, post hoc pairwise comparisons were performed using Bonferroni‐adjusted *t*‐tests. Effect sizes were calculated using partial eta squared ($\eta_{p}^{2}$) to determine the magnitude of observed differences. Statistical significance was set at *p* ≤ 0.05.

## 3 Results

After 8 weeks of intervention, significant reductions in alanine aminotransferase (ALT) were observed in the whey‐supplemented athletes of both modalities. In power‐trained athletes, ALT decreased from 17.5 ± 4.7 UI/L to 15.2 ± 4.1 UI/L (*p* = 0.001; $\eta_{p}^{2}$ = 0.446), while in endurance‐trained athletes it declined from 17.6 ± 4.6 UI/L to 15.4 ± 4.4 UI/L (*p* = 0.001; $\eta_{p}^{2}$ = 0.750). No significant change was found in the power placebo control group (*p* = 0.191; $\eta_{p}^{2}$ = 0.181), whereas the endurance placebo control group showed a significant increase (18.1 ± 3.2 UI/L to 22.2 ± 2.8 UI/L; *p* < 0.001; $\eta_{p}^{2}$ = 0.900) (Table 3, Figure 1).

**TABLE 3:** Effects of whey protein supplementation on hepatic, inflammatory, and muscle damage biomarkers in power‐ and endurance‐trained athletes.

Athletes	Variable	Group	Training period	Mean ± SD	Mean diff.	CI 95%		Comparison	
						Lower	Upper	*p*	$\eta_{p}^{2}$
Power athletes	ALT (UI/L)	WG	Pre	17.5 ± 4.7	2.325	1.081	3.569	0.001	0.446
			Post	15.2 ± 4.1					
		CG	Pré	13.5 ± 3.3	−0.7	−1.821	0.421	0.191	0.181
			Post	14.2 ± 2.8					
	AST (UI/L)	WG	Pre	27.1 ± 5.8	4.2	0.903	7.498	0.015	0.272
			Post	22.9 ± 6.2					
		CG	Pre	22.4 ± 6.2	−1.1	−2.867	0.667	0.193	0.181
			Post	23.5 ± 7.2					
	CRP (mg/L)	WG	Pre	0.35 ± 0.30	0.047	0.003	0.091	0.039	0.206
			Post	0.30 ± 0.33					
		CG	Pre	0.51 ± 0.51	−0.049	−0.108	0.01	0.094	0.281
			Post	0.55 ± 0.56					
	CK (UI/L)	WG	Pre	409 ± 196	46.891	23.245	70.537	0.001	0.476
			Post	362 ± 170					
		CG	Pre	329 ± 228	−15.72	−59.555	28.115	0.438	0.068
			Post	345 ± 211					
Endurance athletes	ALT (UI/L)	WG	Pre	17.6 ± 4.6	2.250	1.626	2.874	0.001	0.750
			Post	15.4 ± 4.4					
		CG	Pre	18,1 ± 3.2	−4.111	−5.228	−2.994	0.001	0.900
			Post	22.2 ± 2.8					
	AST (UI/L)	WG	Pre	21.7 ± 5.2	3.400	2.577	4.223	0.001	0.797
			Post	18.3 ± 4.7					
		CG	Pre	23.0 ± 5.2	−6.556	−7.65	−5.461	0.001	0.960
			Post	29.6 ± 4.8					
	CRP (mg/L)	WG	Pre	0.33 ± 0.30	0.017	−0.029	0.063	0.45	0.03
			Post	0.32 ± 0.29					
		CG	Pre	0.37 ± 0.30	−0.048	−0.118	0.022	0.153	0.238
			Post	0.42 ± 0.32					
	CK (UI/L)	WG	Pre	390 ± 239	55.065	21.335	88.795	0.003	0.381
			Post	335 ± 219					
		CG	Pre	366 ± 138	10.4	−40.771	61.571	0.652	0.027
			Post	355 ± 112					

*Note:* Values represent mean ± standard deviation at pre and post training periods. Mean differences, 95% confidence intervals (CI 95%), and comparison scores (*p* values and partial eta squared) reflect the within group changes over the training period. A *p* value less than 0.05 indicates a statistically significant change.

Abbreviations: $\eta_{p}^{2}$ = partial eta squared; ALT: alanine aminotransferase; AST: aspartate aminotransferase; CG: placebo control group; CK: creatine kinase; CRP: C‐reactive protein; Mean diff.: mean difference; SD: standard deviation; WG: whey group.

**FIGURE 1:**
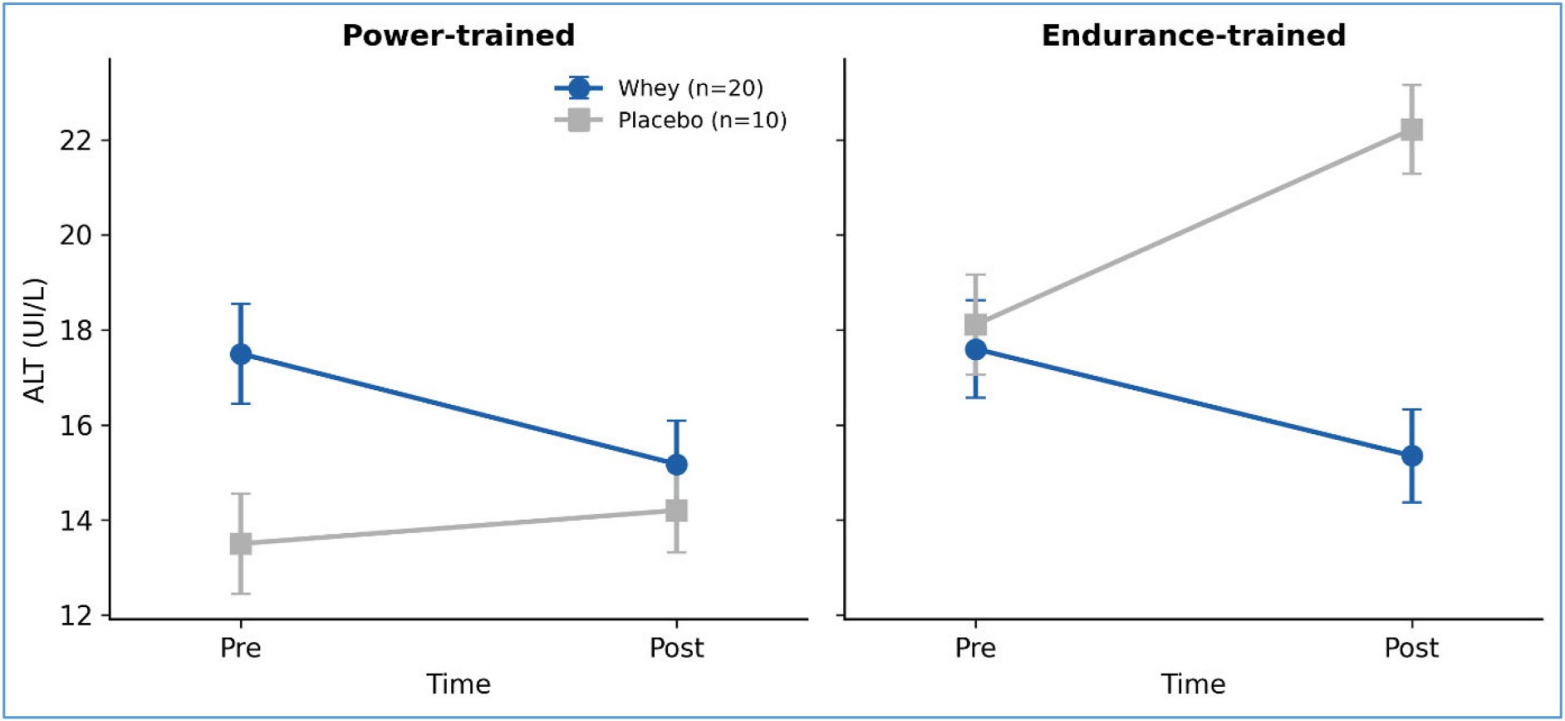
Time × Training Modality × Supplementation interaction for serum ALT. Mean (± SEM) serum alanine aminotransferase (ALT, UI/L) values before (Pre) and after (Post) the 8‐week supplementation period, plotted separately for power‐trained (left panel) and endurance‐trained (right panel) athletes. Blue circles = whey protein group; gray squares = placebo group. The three‐way interaction (Time × Training modality × Supplementation) was statistically significant (*F* (1,55) = 9.51, *p* = 0.003, $\eta_{p}^{2}$ = 0.147), reflecting a decline in ALT in whey‐supplemented athletes of both modalities, contrasted with a marked increase in the endurance placebo group. *n* = 20 (Power‐Whey), 10 (Power‐Placebo), 20 (Endurance‐Whey), 9 (Endurance‐Placebo).

Aspartate aminotransferase (AST) also decreased significantly in the supplemented athletes (power: 27.1 ± 5.8 UI/L to 22.9 ± 6.2 UI/L; *p* = 0.015; $\eta_{p}^{2}$ = 0.272; endurance: 21.7 ± 5.2 U/L to 18.3 ± 4.7 UI/L; *p* = 0.001; $\eta_{p}^{2}$ = 0.797), whereas no change was detected in the power placebo control group (*p* = 0.193; $\eta_{p}^{2}$ = 0.181). The endurance placebo control group displayed a marked increase (23.0 ± 5.2 UI/L to 29.6 ± 4.8 UI/L; *p* < 0.001; $\eta_{p}^{2}$ = 0.960) (Table 3). Because AST is expressed in both hepatic and skeletal‐muscle tissue, its post‐exercise decline likely reflects, at least in part, attenuated skeletal muscle membrane leakage rather than a purely hepatic effect (see Section 4.1 for further discussion).

C‐reactive protein (CRP) changes were generally modest across groups. A statistically significant within‐group reduction was observed in the supplemented power‐trained group (0.35 ± 0.30 mg/L to 0.30 ± 0.33 mg/L; *p* = 0.039; $\eta_{p}^{2}$ = 0.206), which remained significant after Bonferroni adjustment for the number of pairwise comparisons performed. The supplemented endurance group showed only a minor, non‐significant change (0.33 ± 0.30 mg/L to 0.32 ± 0.29 mg/L; *p* = 0.450; $\eta_{p}^{2}$ = 0.030). Conversely, placebo control groups showed slight increases, most pronounced in endurance athletes (0.37 ± 0.30 mg/L to 0.42 ± 0.32 mg/L; *p* = 0.153; $\eta_{p}^{2}$ = 0.238).

Creatine kinase (CK), modifier en vert the study's primary muscle‐damage marker, also declined significantly with whey supplementation in both training modalities. In power‐trained athletes, CK decreased from 409 ± 196 UI/L to 362 ± 170 UI/L (*p* = 0.001; $\eta_{p}^{2}$ = 0.476), while in endurance‐trained athletes it declined from 390 ± 239 UI/L to 335 ± 219 UI/L (*p* = 0.003; $\eta_{p}^{2}$ = 0.381). No significant change was observed in the power placebo control group (329 ± 228 UI/L to 345 ± 211 UI/L; *p* = 0.438; $\eta_{p}^{2}$ = 0.068), and the endurance placebo control group likewise showed no significant change (366 ± 138 UI/L to 355 ± 112 UI/L; *p* = 0.652; $\eta_{p}^{2}$ = 0.027) (Table 3, Figure 2).

**FIGURE 2:**
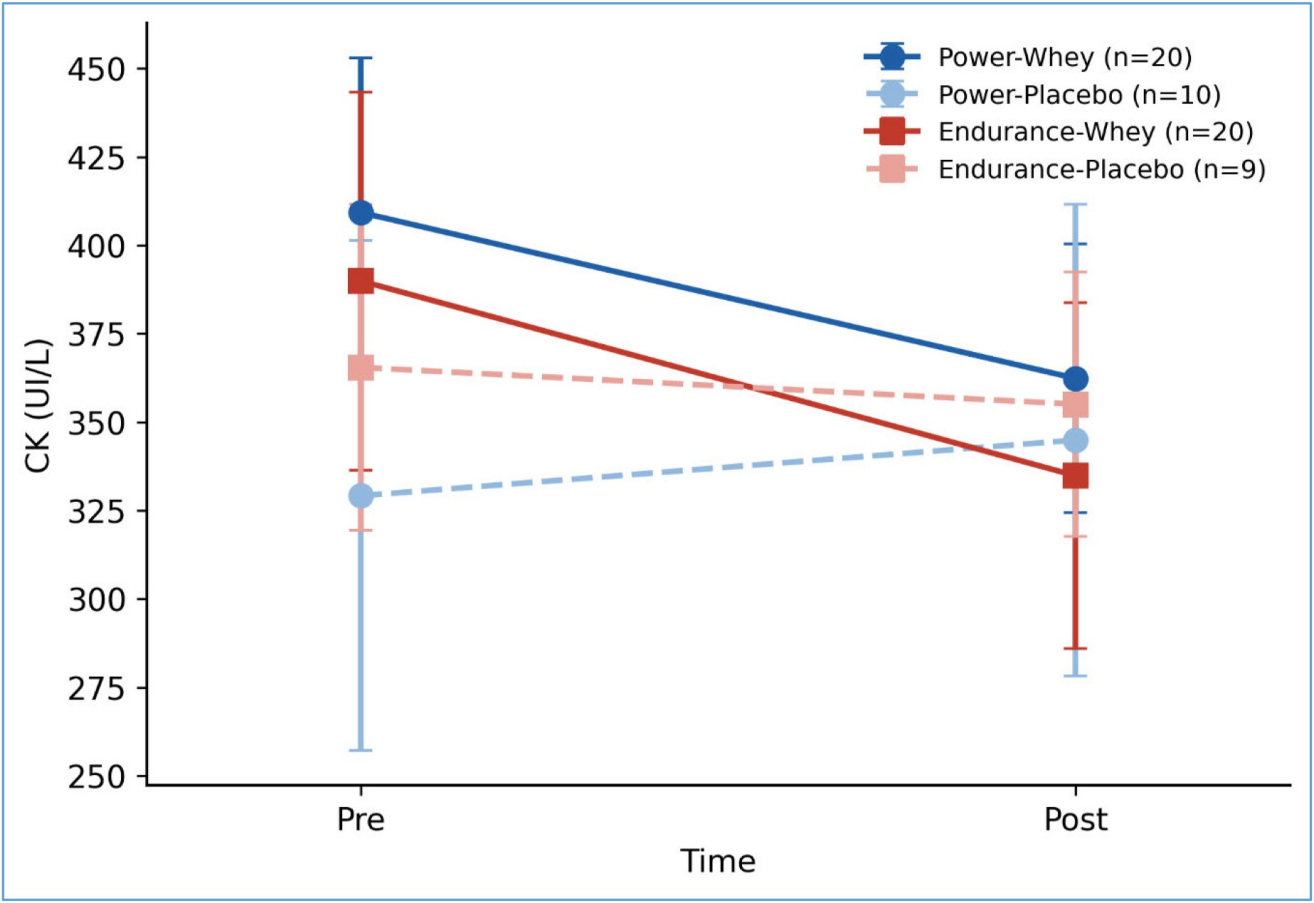
Longitudinal changes in serum creatine kinase (CK) by training modality and supplementation group. Mean (±SEM) serum creatine kinase (CK, UI/L) values before (Pre) and after (Post) the 8‐week supplementation period, for the four training modality × supplementation subgroups: Power‐Whey (*n* = 20, solid blue), Power‐Placebo (*n* = 10, dashed light blue), Endurance‐Whey (*n* = 20, solid red), Endurance‐Placebo (*n* = 9, dashed pink). No statistically significant Time × Training modality × Supplementation interaction was observed (*F* (1,55) = 0.26, *p* = 0.610, $\eta_{p}^{2}$ = 0.005); CK declined in both whey‐supplemented subgroups regardless of training modality, while placebo subgroups remained comparatively stable.

## 4 Discussion

The present study demonstrates that 8 weeks of whey protein supplementation attenuated exercise‐induced increases in biomarkers of hepatic stress (ALT, AST), muscle damage (CK), and inflammation (CRP), with a statistically confirmed modality‐specific interaction (Time × Supplementation × Training modality) for ALT only; a significant main effect of supplementation across both training modalities was also observed for AST, whereas for CK and CRP, significant declines were confined to specific within‐group comparisons (Table 3) without a significant omnibus main effect of supplementation (Table 4). Supplementation appeared to provide physiological protection and support recovery processes, including in power‐trained athletes, though this benefit was not statistically restricted to this group except for ALT.

**TABLE 4:** Within‐subjects contrast analysis: Main effects of time (pre–post), training modality, supplementation, and interactions effects.

Outcomes	Pre–Post	Pre–post *x* TM	Pre–post *x* suppl	Pre–post *x* TM *x* suppl
ALT (UI/L)	*F* (1,55) = 40.44; *p* < 0.001; $\eta_{p}^{2}$ = 0.424	*F* (1,55) = 14.65; *p* < 0.001; $\eta_{p}^{2}$ = 0.210	*F* (1,55) = 66.77; *p* < 0.001; $\eta_{p}^{2}$ = 0.548	*F* (1,55) = 9.51; *p* = 0.003; $\eta_{p}^{2}$ = 0.147
AST (UI/L)	*F* (1,55) = 19.82; *p* < 0.001; $\eta_{p}^{2}$ = 0.265	*F* (1,55) = 7.23; *p* = 0.010; $\eta_{p}^{2}$ = 0.116	*F* (1,55) = 31.53; *p* < 0.001; $\eta_{p}^{2}$ = 0.364	N.S. (*F* (1,55) = 3.57; *p* = 0.064; $\eta_{p}^{2}$ = 0.061)
CRP (mg/L)	N.S. (*F* (1,55) = 2.34; *p* = 0.132; $\eta_{p}^{2}$ = 0.041)	N.S. (*F* (1,55) = 0.00; *p* = 0.977; $\eta_{p}^{2}$ < 0.001)	N.S. (*F* (1,55) = 2.97; *p* = 0.090; $\eta_{p}^{2}$ = 0.051)	N.S. (*F* (1,55) = 0.36; *p* = 0.552; $\eta_{p}^{2}$ = 0.006)
CK (UI/L)	N.S. (*F* (1,55) = 0.25; *p* = 0.621; $\eta_{p}^{2}$ = 0.004)	N.S. (*F* (1,55) = 0.82; *p* = 0.369; $\eta_{p}^{2}$ = 0.015)	N.S. (*F* (1,55) = 3.15; *p* = 0.082; $\eta_{p}^{2}$ = 0.054)	N.S. (*F* (1,55) = 0.26; *p* = 0.610; $\eta_{p}^{2}$ = 0.005)

*Note:* Pre–post *x* TM: Pre–post *x* Training Modality; Pre–post *x* Suppl: Pre–post *x* Supplementation; Pre–post *x* TM *x* Suppl: Pre–post *x* training modality *x* Supplementation; ALT: alanine aminotransferase; AST: aspartate aminotransferase; CRP: C‐reactive protein; CK: creatine kinase; N.S: Not significant.

### 4.1 Hepatic Enzymes (ALT, AST)

Significant reductions in ALT and AST were observed in supplemented athletes regardless of training modality, suggesting hepatocellular protection. These findings are consistent with Kim et al. 2023, who reported beneficial effects of whey protein supplementation on liver enzyme changes during resistance exercise, supporting the notion of reduced hepatic stress during training. These mechanisms may involve whey's antioxidant potential and its ability to preserve cellular integrity under metabolic stress (Devries and Phillips 2015; Phillips 2016; Tipton and Wolfe 2004). Whey proteins are rich in bioactive peptides and cysteine, a precursor of glutathione, a major intracellular antioxidant (Price et al. 2022), which can neutralize reactive oxygen species generated during high‐intensity exercise. Previous work (Peake et al. 2017) also highlights whey's potential to limit hepatic and muscular damage. In contrast, ALT increased markedly in the endurance placebo control group, possibly reflecting uncompensated oxidative and metabolic stress in the absence of nutritional support. It is important to note that while ALT is relatively liver‐specific, AST is also abundantly expressed in skeletal and cardiac muscle tissue; following high‐intensity training, elevations and subsequent reductions in serum AST frequently reflect sarcolemmal membrane permeability changes associated with skeletal muscle micro‐damage rather than isolated hepatocellular pathology (Pettersson et al. 2008). The close parallel between the post‐supplementation declines in CK and in AST/ALT observed in the present study (Table 3) suggests that at least part of the reduction in circulating transaminases is a secondary consequence of attenuated skeletal muscle breakdown, rather than evidence of a hepatoprotective action acting independently of skeletal muscle protection. Disentangling the hepatic versus muscular origin of the AST signal would require complementary markers with greater tissue specificity (e.g., gamma‐glutamyltransferase for hepatic origin, or myoglobin/skeletal troponin for muscular origin), which were not measured in the present study and are recommended for future work (Brancaccio et al. 2010).

### 4.2 Muscle Damage (CK)

Whey supplementation significantly reduced CK levels in both endurance and power‐trained athletes, indicating a protective effect on muscle fibers. This aligns with Tang et al. (2009) and with the meta‐analysis by Poulios et al. (2019), which reported that animal protein supplementation can reduce post‐exercise CK elevations by up to 40%. The rapid absorption of whey and its high content of branched‐chain amino acids (BCAAs), especially leucine and isoleucine, contribute to stimulating muscle protein synthesis, limiting proteolysis, and accelerating structural repair (Xu et al. 2025). Hansen et al. (2015) similarly observed reduced muscle damage markers and enhanced recovery following protein hydrolysate ingestion in elite runners. As the three‐way interaction (Time × Supplementation × Training modality) for CK was not statistically significant (Table 4), and the overall Pre‐post × Supplementation main effect was also non‐significant (F(1,55) = 3.15, *p* = 0.082; Table 4), this CK‐lowering effect appears confined to the whey‐supplemented subgroups specifically (Table 3) rather than reflecting either a significant omnibus main effect of supplementation or a power‐specific benefit.

### 4.3 Inflammatory Response (CRP)

Although CRP changes were small and non‐significant overall, power‐trained athletes in the supplemented group showed a significant within‐group reduction despite high‐intensity training, although this reduction did not correspond to a statistically significant differential effect across training modalities (see Section 3, and Table 4, where the three‐way interaction for CRP was non‐significant). This finding suggests a possible modulatory effect of whey on systemic inflammation, consistent with Peake et al. (2017) and Mohammadi et al. (2025), who reported reductions in pro‐inflammatory cytokines following whey intake. Buckley et al. (2010) observed similar CRP attenuation in athletes undergoing eccentric exercise. The mechanism may involve cysteine‐driven glutathione synthesis, which mitigates oxidative and inflammatory stress (Farahmandpour et al. 2025). Overall, in light of the non‐significant omnibus interaction, this CRP finding should be regarded as a modest within‐group trend warranting confirmation in an adequately powered sample, rather than as firm evidence of modality‐dependent anti‐inflammatory action.

### 4.4 Differences Between Endurance and Power‐Trained Athletes

Our findings indicate that training modality significantly influenced supplementation outcomes specifically for ALT (significant three‐way interaction, *p* = 0.003). As highlighted by Phillips and Van Loon (2011), metabolic adaptations and nutrient responsiveness vary with the nature of the training stimulus. Endurance trained athletes often present with cumulative oxidative stress but may exhibit lower levels of chronic inflammation (Peake et al. 2017), which could partly explain the smaller effect on CRP. Power‐trained athletes, exposed to greater mechanical loading and eccentric muscle actions, may derive more benefit from whey's anabolic and anti‐catabolic properties (Churchward‐Venne et al. 2012), a hypothesis supported for ALT in the present data but that will require replication with adequately powered samples for AST, CK, and CRP before being generalized.

### 4.5 Practical Implications

These data support the integration of whey protein supplementation into individualized recovery protocols, particularly in sports involving high muscular strain. Tailoring supplementation strategies to the specific metabolic demands of each discipline could optimize recovery kinetics and minimize biochemical markers of overreaching.

### 4.6 Limitations and Future Directions

Although the study was adequately powered to detect primary interaction effects (group *x* time), the sample size may still limit the generalizability (external validity) of our findings regarding secondary outcomes, specific inflammatory responses like CRP, and direct field performance. This power calculation, however, was based on a two‐way within‐between model targeting the primary Time × Supplementation interaction (88%) and does not account for the additional loss of power inherent to the three‐way Time × Supplementation × Training modality interaction reported in Table 4, for which the risk of a Type II error cannot be excluded; the a priori calculation also relied on a conventional medium effect size (*f* = 0.25) rather than a pilot‐derived estimate specific to this cohort. To address these limitations, we suggest that future large‐scale investigations focus on dose‐response effects, optimal timing of supplement ingestion, and long‐term impacts on recovery and athletic performance. Additionally, the integration of a longitudinal analysis of biomarkers could significantly improve the personalization of nutritional strategies in competitively trained athletes. Several additional methodological limitation warrant mention. First, baseline dietary intake, including habitual protein consumption (g/kg/day), was not systematically quantified; therefore, it cannot be excluded that part of the observed biochemical changes reflect correction of an underlying baseline protein deficit in some participants rather than a specific ergogenic effect of whey, and the carbohydrate‐based placebo may itself have exerted an insulin‐mediated anti‐proteolytic effect. Second, the power‐trained cohort combined athletes from mechanically and metabolically heterogeneous disciplines (throwers, jumpers, and sprinters), producing substantial intra‐group variance in body mass (± 11.9 kg in the placebo subgroup, Table 1); because CK and transaminase activity correlate with active muscle mass and mechanical loading, this heterogeneity may have introduced statistical noise that masked or distorted true supplementation effects. Third, the inclusion criterion of ≥ 6 months of regular training corresponds to a “trained/developmental” rather than strictly “elite” classification under current participant‐classification frameworks (McKay et al. 2022); although all athletes competed at national or international level, findings should not be generalized to athletes with many years of specialized high‐volume training without further verification. Fourth, this study included only male athletes, limiting the generalizability of findings to female populations, who differ in hormonal profile and inflammatory response to exercise.

## 5 Conclusions

Eight weeks of daily whey protein post exercise supplementation (30 g) reduced ALT and AST levels across both training modalities, produced within‐group declines in CK regardless of modality, and led to a within‐group stabilization of CRP in power‐trained athletes, with a statistically confirmed modality‐specific benefit restricted to ALT. These results support the potential role of whey protein intake as a recovery‐enhancing nutritional intervention, particularly in sports with high anaerobic and mechanical demands. Given the influence of training modality on hepatic‐marker responses to supplementation, further adequately powered studies are recommended to confirm whether modality‐specific strategies are warranted for other biomarkers. Further research incorporating performance metrics and oxidative stress markers is needed.

## Author Contributions

M.B.M., F.Z., and M.L. contributed to the study's conception, design, interpretation of results, and manuscript preparation (original draft and revisions). M.B.M., M.M., A.B., and H.T. conducted the experimental work and performed biochemical analyses. F.Z. was responsible for statistical analysis. K.W., T.R., E.B., and B.K. contributed to conceptualization, supervision, and critical revision of the manuscript. K.W., T.R., and B.K. additionally handled editing, project administration, and funding acquisition. All authors reviewed and approved the final version of the manuscript and consented to its submission.

## Funding

The authors gratefully acknowledge the Laboratory of Clinical Biochemistry and Hormonology, Pasteur Institute of Tunis for all biochemistry analysis and for its financial support and collaboration in this study.

## Conflicts of Interest

The authors declare no conflicts of interest.

## Data Availability

The data that support the findings of this study are available on request from the corresponding author. The data are not publicly available due to privacy or ethical restrictions.
